# A fully human neutralizing monoclonal antibody targeting a highly conserved epitope of the human cytomegalovirus glycoprotein B

**DOI:** 10.1371/journal.pone.0285672

**Published:** 2023-05-16

**Authors:** Miwa Okamoto, Rika Kurino, Ryu Miura, Kenzo Takada

**Affiliations:** Sapporo Laboratory, EVEC, Inc., Sapporo, Hokkaido, Japan; University of South Florida, UNITED STATES

## Abstract

Human cytomegalovirus causes severe diseases in children (by congenital infection) and immunocompromised patients. Treatment with antiviral agents, such as ganciclovir, is limited by their toxicity. In this study, we investigated the effectiveness of a fully human neutralizing monoclonal antibody to inhibit human cytomegalovirus infection and viral cell-to-cell spread. We isolated a potent neutralizing antibody, EV2038 (IgG1 lambda), targeting human cytomegalovirus glycoprotein B using Epstein-Barr virus transformation. This antibody inhibited human cytomegalovirus infection by all four laboratory strains and 42 Japanese clinical isolates, including ganciclovir-resistant isolates, with a 50% inhibitory concentration (IC_50_) ranging from 0.013 to 0.105 μg/mL, and 90% inhibitory concentration (IC_90_) ranging from 0.208 to 1.026 μg/mL, in both human embryonic lung fibroblasts (MRC-5) and human retinal pigment epithelial (ARPE-19) cells. Additionally, EV2038 prevented cell-to-cell spread of eight clinical viral isolates, with IC_50_ values ranging from 1.0 to 3.1 μg/mL, and IC_90_ values ranging from 13 to 19 μg/mL, in ARPE-19 cells. EV2038 recognized three discontinuous sequences on antigenic domain 1 of glycoprotein B (amino acids 549–560, 569–576, and 625–632), which were highly conserved among 71 clinical isolates from Japan and the United States. Pharmacokinetics study in cynomolgus monkeys suggested the potential efficacy of EV2038 *in vivo*, the concentration of which in serum remained higher than the IC_90_ values of cell-to-cell spread until 28 days after intravenous injection of 10 mg/kg EV2038. Our data strongly support EV2038 as a promising candidate and novel alternative for the treatment of human cytomegalovirus infection.

## Introduction

Human cytomegalovirus (HCMV) is a ubiquitously distributed beta-herpesvirus, with the prevalence of infection ranging from 60% to almost 100%, according to age and socioeconomic status [1–3]. HCMV infection is usually asymptomatic; however, it is also a significant risk factor for serious HCMV disease in certain groups, including babies and immunocompromised patients [2]. There remains no effective vaccine [4], and first-line treatment with current antiviral agents, such as ganciclovir, is often restricted by toxicity and the induction of resistance [5–7].

As an alternative treatment option, the use of hyperimmune globulin (HIG), purified from human plasma with high-titer HCMV antibody, has been proposed. HIG can be used safely and effectively in pregnant women, fetuses [8, 9], and transplant recipients in combination with antiviral agents [10–12]. However, polyclonal HIG is difficult to standardize and less specific than monoclonal antibodies (mAbs). Thus, human mAbs specifically neutralizing viral infections are in development [13].

HCMV encodes multiple envelope glycoproteins to infect a large variety of cell types [14]. The major antigen epitopes targeted by neutralizing mAbs are concentrated in glycoprotein B (gB) and glycoprotein H (gH). During viral entry, gB mediates membrane fusion in combination with the gH/gL/gO trimer in fibroblasts and the gH/gL/pUL128/130/131 pentamer in epithelial and endothelial cells [15–18]. gB is the most immunodominant envelope glycoprotein. It has five antigenic domains (AD-1 to AD-5), with neutralizing epitopes at AD-1, AD-2 site I, AD-4, and AD-5 [19–22]. Neutralizing mAbs targeting AD-2 site I and AD-4 have been developed [23–25], including TI-23 (Teijin Pharma), TCN-202 (Theraclone Sciences), TRL345 (Trellis Bioscience), and LJP538 in CSJ148 (Novartis). Anti-gH mAbs have also been developed [23], including MSL-109 (Novartis) and MCMV5322A in RG7667 (Genentech) targeting gH/gL and LJP539 in CSJ148 (Novartis) and MCMV3068A in RG7667 (Genentech) targeting gH-pentamer. mAbs targeting gH-pentamer are often combined with mAbs targeting other regions, because they lack neutralizing activity in fibroblasts [26–28]. Several candidate mAbs have entered clinical trials. However, none have demonstrated efficacy to date [29–36].

We obtained a novel clinical candidate mAb, EV2038, targeting AD-1 of HCMV gB [37]. AD-1 is the most immunodominant epitope, with 97% of HCMV-seropositive individuals developing antibodies against this epitope [22, 38]. AD-1 is considered to play an essential role in gB oligomerization, which is required for gB folding and infectious virus assembly [39]. Although only 2% of AD-1-specific antibodies neutralize HCMV [22], EV2038 was successfully isolated as a potent neutralizing mAb from a healthy donor by Epstein-Barr virus (EBV) transformation. In this study, we investigated the effectiveness of EV2038 in neutralizing HCMV *in vitro*. EV2038 effectively inhibited HCMV infection and viral cell-to-cell spread in all laboratory and clinical isolates tested. The antibody recognized discontinuous but highly conserved sequences of AD-1, which may contribute to the potent neutralizing activity of EV2038.

## Materials and methods

### EV2038 isolation

EV2038 was isolated from a donor whose serum antibodies were highly specific for recombinant AD-1 of HCMV gB. Peripheral blood and written informed consent from all donors were obtained under study protocols approved by the Ethics Committee of the EVEC Internal Review Board (EVEC-2006-045). B lymphocytes in peripheral blood mononuclear cells of the donor were infected with EBV, as previously described [40]. EBV-transformed cells were suspended in RPMI 1640 medium (Invitrogen) supplemented with 10% fetal bovine serum (FBS) and 2 mM L-glutamine, and seeded in 96-well microplates in the presence of irradiated allogeneic feeder cells. The cells were cultured at 37°C in a humidified atmosphere containing 5% CO_2_ for 2–3 weeks. Antibodies in undiluted culture supernatants were screened for their specificity to recombinant AD-1 protein using enzyme-linked immunosorbent assay (ELISA), as described under the ELISA subheading. Cells in positive wells were seeded for limiting dilution and screened as described above until a positive cell was cloned.

Total RNA was extracted from cloned cells using the QIAamp RNA Blood Mini Kit (Qiagen). cDNA was synthesized with oligo dT primers using the Cells-to-cDNA Kit (Invitrogen), according to the manufacturer’s protocol. Full-length genes of heavy and light chains were amplified by PCR using KOD-plus DNA polymerase (Toyobo) and primers against translation initiation (5ʹ-end) and termination (3ʹ-end) sites. The secondary PCR was performed using primers introducing a pcDNA3.1 directional TOPO expression vector (Invitrogen) cloning site. The PCR products were ligated into the expression vector and transformed into *Escherichia coli* (*E*. *coli*) TOP10 cells (Invitrogen). After plasmid isolation, heavy and light chains were sequenced using a 3130 Genetic Analyzer (Applied Biosystems). Complementarity-determining regions (CDRs) were analyzed according to Kabat’s method (www.bioinf.org.uk: Dr. Andrew C. R. Martin’s Group; Antibodies: General Information).

For antibody purification, heavy and light chain genes were assembled on a single vector using GS system pEE GS expression vectors (Lonza Biologics). The expression plasmid was transfected into CHO-K1SV cells (Lonza Biologics), followed by the selection of high-expressing clones, according to the manufacturer’s protocol. Antibodies in culture supernatants were purified by affinity chromatography using a prepacked HiTrap rProtein A Fast Flow column (GE Healthcare).

### ELISA

Recombinant AD-1 protein from HCMV gB was used for ELISA. A full sequence of AD-1 protein (strain AD169, amino acids, 541–658; UniProtKB accession number: P06473) was fused with the glutathione S-transferase tag of a pGEX4T vector and expressed in *E*. *coli* BL21 (DE3) cells. The cells were induced with IPTG (0.5 mM) and harvested 3 hours post-induction. The cell pellets were washed with 1% Triton X-100 and solubilized with 0.5% sarkosyl to obtain the recombinant protein. Recombinant AD-1 protein (40.7 kDa) was purified according to the protocol of “Electropurification of Proteins” (Rout Lab Protocol, 2007, https://lab.rockefeller.edu/rout/assets/file/protocols/Electropurification.pdf). Briefly, the recombinant protein was separated by sodium dodecyl sulfate–polyacrylamide gel electrophoresis (SDS-PAGE) and stained with KCl (0.25 M). The protein band was cut and electrically eluted into a dialysis tubing filled with 0.5 X SDS-PAGE buffer. The protein was dialyzed with phosphate-buffered saline (PBS (-)).

ELISA 96-well plates (NUNC) were coated with 5 μg/mL recombinant AD-1 protein in PBS (-) at 4°C overnight. After washing with PBS (-) containing 0.1% Tween 20, the plates were blocked with 20% N101 (NOF Corp.) for 2 hours at room temperature. After the blocking solution was removed, purified antibodies diluted in 10% FBS–RPMI 1640 were added and incubated for 1 hour at room temperature. Binding antibodies were detected using horseradish peroxidase-labeled anti-human IgG (MBL) and TMB substrate (Sigma).

### Neutralization assay

#### Cells and viruses

Human embryonic lung fibroblast (MRC-5) and human retinal pigment epithelial (ARPE-19) cells were purchased from the European Collection of Cell Cultures and the American Type Culture Collection, respectively. MRC-5 cells were cultured in 10% FBS–Eagle’s Minimal Essential Medium, whereas ARPE-19 cells were cultured in 10% FBS–Dulbecco’s Modified Eagle’s Medium (DMEM) supplemented with Ham’s F-12 nutrient mixture.

HCMV laboratory strains (AD169, Towne, and Davis) and a low-passage clinical isolate (Merlin) were purchased from the American Type Culture Collection. Japanese clinical isolates were provided by the Department of Microbiology, Fukushima Medical University School of Medicine, Fukushima, Japan; the Virus Research Center, Clinical Research Division, Sendai Medical Center, Sendai, Japan; and Kagoshima University Graduate School of Medical and Dental Sciences, Kagoshima, Japan. Viruses were propagated in MRC-5 or ARPE-19 cells in 2% FBS medium and then stored in 35% sorbitol at –80°C until use. Virus propagation and titration were performed before the assay.

#### Inhibition assay of virus infection

The neutralizing activity of EV2038 against HCMV infection was determined by plaque reduction assay. Because complements did not influence the neutralizing titers of EV2038 in a preliminary assay (S1 Fig), further neutralization assays were performed without complements. HCMV (AD169, Towne, Davis, Merlin, or 42 Japanese clinical isolates) was mixed with 2-fold serial dilutions of antibodies in 2% FBS medium for 1 hour at 37°C. Four ganciclovir-resistant isolates (93R, 99-DZN, H12-42, and SI; S2 Fig), with a 50% inhibitory concentration (IC_50_) of ganciclovir greater than 12 μmol/L [41], were selected in a preliminary assay. The final antibody concentrations were 0.0012–10 μg/mL for EV2038, 0.12–1,000 μg/mL for CytoGam (CMV hyperimmune globulin; CSL Behring), and 10 μg/mL for the negative control (anti-hCD20-hIgG1; Invitrogen). For clinical isolate assays, the concentration of EV2038 ranged from 0.0024 to 40 μg/mL with 4-fold serial dilutions. A virus mixture without mAb was prepared as a vehicle control. The virus–antibody mixture (100 plaque-forming units per well) was added to MRC-5 or ARPE-19 monolayers. Cells were incubated for 1 hour at 37°C, centrifuged at 600 ×*g* for 40 minutes at room temperature, and further incubated for 1 hour at 37°C. After washing twice, the cells were cultured in fresh 5% FBS medium for 4–11 days at 37°C, until plaques were visible. The cells were fixed in 10% formalin in PBS (-) and stained with 0.025% crystal violet. The number of plaques was counted manually using an inverted light microscope. Neutralizing titers were calculated by subtracting the percentage of vehicle control (the number of plaques with antibodies was divided by the number of plaques without antibodies) from 100%. IC_50_ and the 90% inhibitory concentration (IC_90_) values were calculated using a non-linear regression curve-fitting model with a variable slope (GraphPad Prism 5.04; GraphPad Software Inc.).

#### Inhibition assay of viral cell-to-cell spread

The inhibitory effect of EV2038 on viral cell-to-cell spread was examined in eight Japanese clinical isolates (2009–889, I88-55, I144-44, H12-42, H12-43, H1-10, H1-15, and MDU-1) by a focus expansion assay [42]. The virus (100 plaque-forming units per well) was added to ARPE-19 monolayers. The cells were incubated for 1 hour at 37°C, centrifuged at 600 ×*g* for 40 minutes at room temperature, and further incubated for 6 hours at 37°C. After washing twice, three wells per virus strain were fixed to determine the number of initially infected cells. For viral expansion, 2-fold serial dilutions of EV2038 in 2% FBS–DMEM were added to the cells and overlaid with the same volume of 1.6% methylcellulose in 2% FBS–DMEM. The final EV2038 concentrations ranged from 0.313 to 160 μg/mL (0 μg/mL for the vehicle control). The cells were cultured for 14 days at 37°C. On Day 7, the culture medium was replaced with fresh mAb at each concentration in 2% FBS–DMEM containing 0.8% methylcellulose. The cells were fixed in absolute ethanol. The HCMV-infected cells were detected using mouse anti-CMV immediate-early (IE) 1 and IE2 antibody (Abcam) as the primary antibody and Alexa Fluor 546-labeled anti-mouse IgG antibody (Life Technologies) as the secondary antibody. The number of infected cells was counted manually using a fluorescence microscope and subtracted by the number of initially infected cells. Neutralizing titers were calculated by subtracting the percentage of vehicle control from 100%. IC_50_ and IC_90_ values were calculated using non-linear regression analysis with a sigmoid-E_max_ model (SAS Institute).

### Epitope mapping

Epitope mapping was performed using EV2038 and AD-1 peptides (strain AD169; amino acids, 541–652). In total, 26 peptides of 12 amino acids shifted by four residues were synthesized by a peptide synthesis service (Sigma Genosys). The C-terminus of each peptide was bound to the surface of the derivatized cellulose membrane to form a spot (SPOT method; Sigma Genosys). Peptide spots were analyzed by Western blotting to detect EV2038-binding specificity.

### Surface plasmon resonance

Surface plasmon resonance measurements were performed using a Biacore T200 system (GE Healthcare). Anti-IgG (Fc) antibodies (Human Antibody Capture Kit; GE Healthcare) were immobilized on a CM5 sensor chip (GE Healthcare), according to the manufacturer’s protocol. EV2038 (0.33 μg/mL) was injected and captured on the sensor chip. Various concentrations of recombinant AD-1 were then injected. For the analysis of binding affinity to HCMV virions, 200 μg/mL MSL-109 (anti-HCMV gH/gL) was captured first. In the subsequent injection of crude virus purification sample (strain AD169; purified via ultracentrifugation, 1.6 × 10^8^ plaque-forming units/mL), infectious virions were trapped by the captured MSL-109. Various concentrations of EV2038 were then injected. Kinetic parameters were calculated from the obtained sensorgrams, using a 1:1 binding model (Biacore T200 evaluation software 1.0; GE Healthcare).

### Pharmacokinetics and tissue cross-reactivity studies

EV2038 was administered one or four times to cynomolgus monkeys (male and female, 3−6 years of age). This study was carried out in accordance with the animal welfare bylaws of Shin Nippon Biomedical Laboratories, Ltd., Drug Safety Research Laboratories, which is fully accredited by AAALAC International. The protocols were approved by the Institutional Animal Care and Use Committee (Protocol Numbers: IACUC321-606 and IACUC321-607). Animal husbandry conditions were maintained as follows: 23.5‒27.5°C temperature, 39‒74% humidity, 15 times/hour ventilation, 12 hours/day of artificial light illumination, daily water wash of rooms and cages, and replacement of cages with cleaned and disinfected ones at least once in 4 weeks. A pellet diet was provided to animals once daily, and water was available *ad libitum* from an automatic water supply system. Treats (apple) were supplied twice weekly. Enrichment toys were provided. Animals were observed for clinical signs, including external appearance, behavior, respiration, position, urinary and fecal conditions, and response to stimulation, and for mortality throughout the observation period (dosing day: 4 times daily, non-dosing day: twice daily, and recovery period: once daily). After the final observation, animals were anesthetized by an intravenous injection of sodium pentobarbital (64.8 mg/mL, 0.4 mL/kg) into the tail vain, and euthanized by exsanguination. All efforts were made to minimize suffering.

For the single dose study, EV2038 at doses of 10 and 100 mg/kg in acetic acid buffer (pH 5.0) containing 4.372% (w/v) sorbitol and 0.02% (w/v) polysorbate 80 was intravenously administered to two animals/sex/group via the cephalic vein at 5 mL/minute, and the animals were examined for 4 weeks. The high dose was chosen as a feasible high dose for toxicity studies of biopharmaceuticals based on the highest EV2038 concentration (10 mg/mL) and the maximum dose in cynomolgus monkeys (10 mL/kg). For the repeated dose study, EV2038 at doses of 0, 50, and 100 mg/kg was intravenously administered to three (0 and 50 mg/kg doses) or six (100 mg/kg dose) animals/sex/group once a week for 4 weeks (a total of four injections). The animals were examined over a 4-week dosing period. Half of the animals in the 100 mg/kg dose group were further examined over a 4-week recovery period.

The following observations and examinations were performed: mortality, clinical signs, body weight, food consumption, ophthalmology, electrocardiography, blood pressure, respiratory rate, urinalysis, hematology, blood chemistry, gross pathology, organ weights, histopathology, and anti-EV2038 antibody analysis. Anti-EV2038 antibodies were detected by electrochemiluminescence assay. Animal serum was mixed with biotinylated and ruthenylated EV2038 and incubated at 4°C overnight. The antibody mixture was captured on streptavidin-coated 96-welled plates for 1 hour at room temperature, and the luminescence intensity was measured.

For pharmacokinetic parameters, serum concentrations of EV2038 at multiple time points (pre-dose and 10 minutes, 4/24/72/168 hours, and 14/21/28 days post-dose) were quantified by electrochemiluminescence assay. Animal serum was added to the wells, which were coated with anti-EV2038 idiotype antibody, and incubated for 1 hour at room temperature. Captured EV2038 was detected using biotinylated anti-EV2038 idiotype antibody and Sulfo-tag labeled streptavidin. The luminescence intensity was measured and used to calculate EV2038 concentration using a standard curve fitted with a four-parameter logistic model with 1/Y^2^ weighting. EV2038 concentrations in serum at the 0 mg/kg dose were below the lower limit of quantification (10 ng/mL) throughout the study. Pharmacokinetic parameters were calculated using a non-compartmental model (WinNonlin 5.2 software; Pharsight Corp.).

The tissue cross-reactivity of EV2038 was assessed using immunohistochemistry in the following tissues: adrenal, urinary bladder, blood cells (peripheral blood smears), bone marrow, mammary gland (breast), brain cerebellum, brain cortex, blood vessel (endothelial), eye, fallopian tube, esophagus, gastric antrum, gastric body, duodenum, ileum, colon, heart, kidney, liver, lung, lymph node, ovary, pancreas, parathyroid, parotid, peripheral nerve, pituitary, placenta (human only), prostate, skin, spinal cord, spleen, skeletal muscle, testis, thymus, thyroid, tonsil, ureter, uterus cervix, and uterus endometrium. Cryo-sections from frozen tissues of humans and cynomolgus monkeys (n = 2–3) were fixed in acetone. HCMV-infected and non-infected MRC-5 cells were used as controls. Acetone-fixed tissue slides and controls were stained with biotinylated EV2038 or control human IgG lambda at 2.5, 5.0, and 10.0 μg/mL and examined histopathologically (Covance Laboratories Ltd.). Viable tissues were confirmed by antibody reactivity against vimentin, cytokeratin, CD71 (human only), and von Willebrand factor. The absence of autolysis was confirmed using hematoxylin and eosin-stained slides.

## Results

### Neutralizing activity of EV2038 *in vitro*

We isolated a total of 14 AD-1-specific mAbs from five donors pre-selected for the presence of serum antibodies bound to recombinant AD-1. EV2038 (IgG1 lambda) was selected as the lead candidate, owing to its high neutralizing activity. The amino acid sequences of the CDRs of EV2038, according to Kabat’s definition, are listed in Table 1.

**Table 1:** Amino acid sequences of the CDRs of EV2038 according to Kabat’s definition.

	CDR1	CDR2	CDR3
**Heavy chain**	GYYWG	EINHSGSANSNPSLKS	VTRDLEWIPGDYYMDV
**Light chain**	SGSLSNIGTNYVY	KNNQRPS	AAWDDSLNGYV

CDR, complementarity-determining region

**Fig 1:**
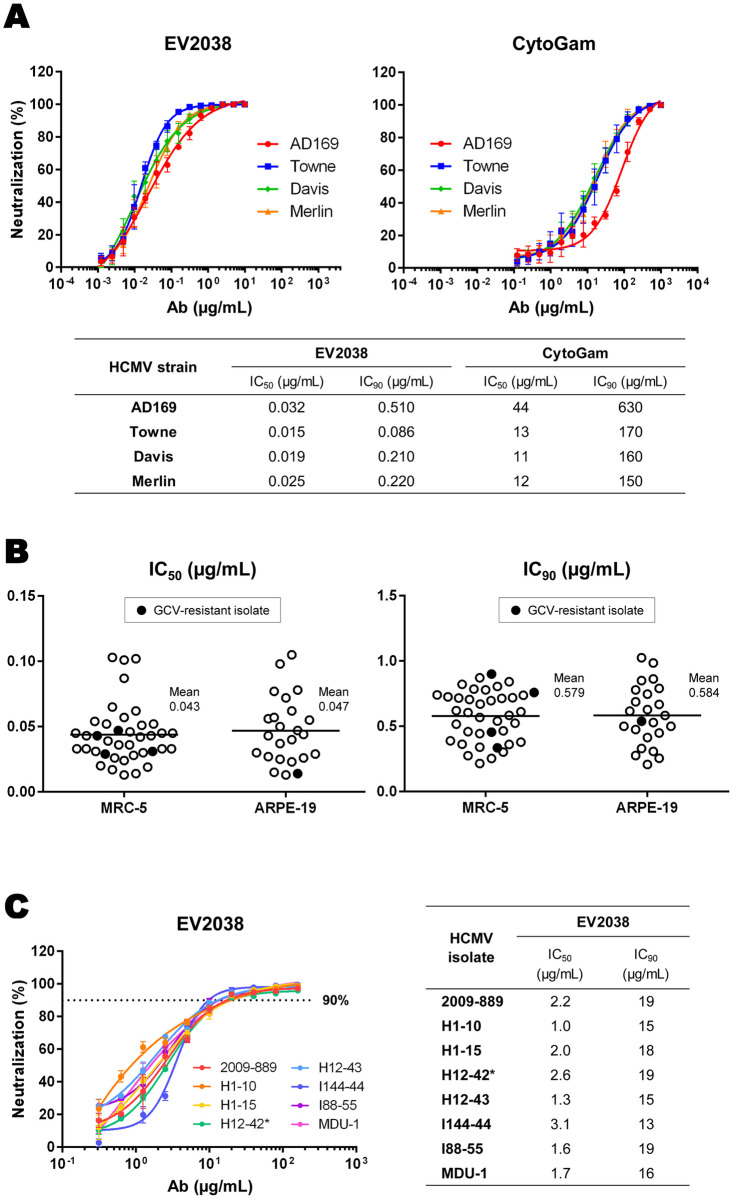
Neutralizing activity of EV2038 *in vitro*. **(A)** Neutralizing activity of EV2038 and CytoGam against four strains of human cytomegalovirus (AD169, Towne, Davis, and Merlin) in MRC-5 cells. Data represent geometric mean with error bars indicating the SD of four independent experiments performed in triplicate. **(B)** The 50% (left) and 90% (right) inhibitory concentrations against 42 Japanese clinical isolates in MRC-5 (39 isolates) and ARPE-19 (24 isolates) cells. Filled symbols indicate ganciclovir-resistant isolates. Data represent the mean of triplicates from one experiment. **(C)** Inhibitory activity of EV2038 against cell-to-cell spread of eight Japanese clinical isolates in ARPE-19 cells. Data represent mean with error bars indicating the SD of triplicates from one experiment. The asterisk indicates a ganciclovir-resistant isolate.

EV2038 neutralized all four laboratory strains, with IC_50_ values ranging from 0.015 to 0.032 μg/mL, and IC_90_ values ranging from 0.086 to 0.510 μg/mL (Fig 1A). CytoGam neutralized the HCMV strains at higher concentrations than EV2038, with IC_50_ values ranging from 11 to 44 μg/mL, and IC_90_ values ranging from 150 to 630 μg/mL. Anti-hCD20-hIgG1, a negative control mAb, showed no inhibition against any strain at a concentration of 10 μg/mL.

The efficacy of EV2038 against HCMV infection was further determined in 42 clinical isolates, including four ganciclovir-resistant isolates. EV2038 neutralized all 39 isolates in MRC-5 cells, with IC_50_ values ranging from 0.013 to 0.103 μg/mL, and IC_90_ values ranging from 0.215 to 0.901 μg/mL (Fig 1B). EV2038 also neutralized all 24 isolates in ARPE-19 cells, with IC_50_ values ranging from 0.013 to 0.105 μg/mL, and IC_90_ values ranging from 0.208 to 1.026 μg/mL (Fig 1B). No statistically significant differences were observed between the two cell types (unpaired *t*-test with Welch’s correction, two-tailed; p = 0.598 (IC_50_), p = 0.922 (IC_90_)).

Additionally, EV2038 effectively prevented the cell-to-cell spread of all HCMV clinical isolates, with IC_50_ values ranging from 1.0 to 3.1 μg/mL, and IC_90_ values ranging from 13 to 19 μg/mL (Fig 1C).

### Epitope mapping

**Fig 2:**
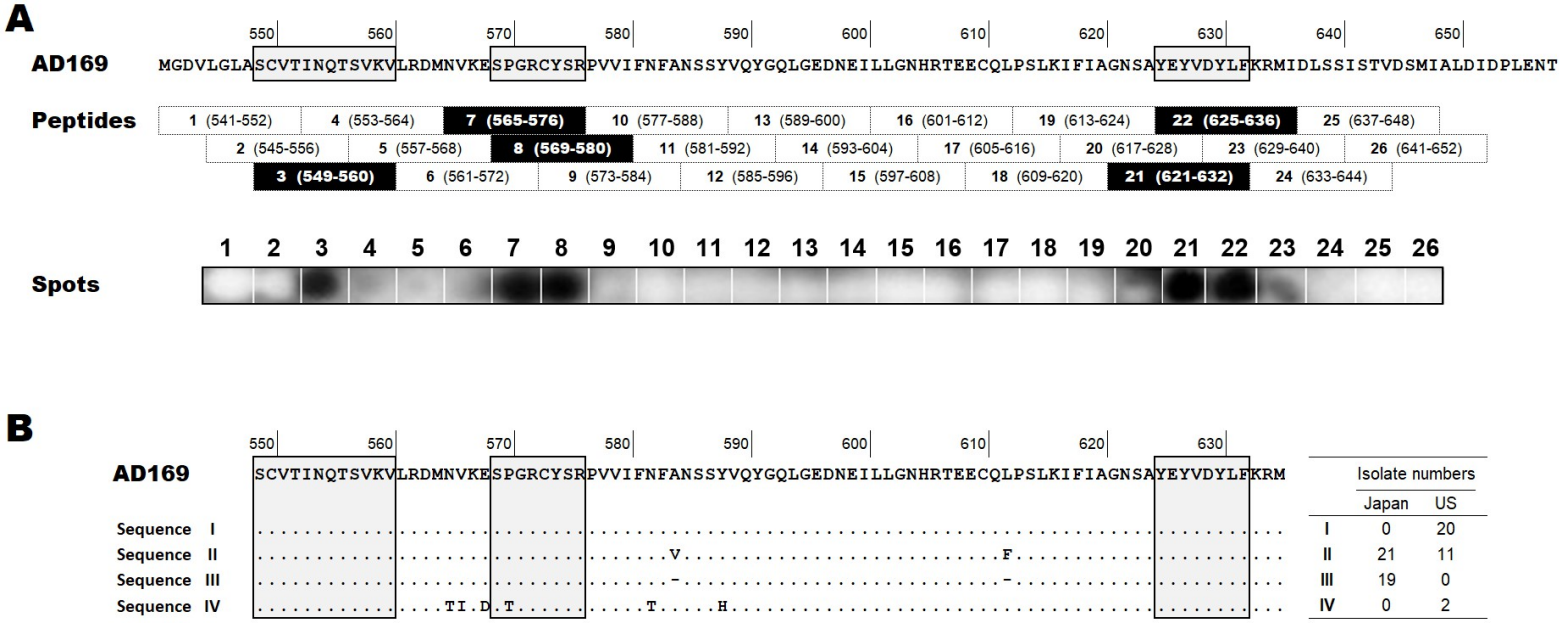
Epitope mapping of EV2038 on antigenic domain 1 (amino acids, 541–652). The amino acid numbers start at the methionine initiation codon of human cytomegalovirus glycoprotein B (strain AD169). The predicted EV2038-binding sequences are surrounded by a frame. **(A)** Positions of 26 antigenic domain 1 peptides and EV2038-binding spots detected by Western blotting. **(B)** Alignment of amino acid sequences of antigenic domain 1 among Japanese (n = 40) and United States (n = 33) clinical isolates. Isolates were divided into four groups (I–IV) according to differences in the sequence, representing isolated numbers of each group in the right-hand table. Dots and dashes represent the same and deleted amino acids compared with the laboratory strain, AD169, respectively.

Antigenic sites of AD-1 recognized by EV2038 were examined by Western blotting of peptides. EV2038 binding was observed in five of 26 peptides spanning AD-1, and three discontinuous sequences of AD-1 were found to be recognized by EV2038 (Fig 2A). The amino acid sequences of the three discontinuous sequences were SCVTINQTSVKV (amino acids, 549–560), SPGRCYSR (amino acids, 569–576), and YEYVDYLF (amino acids, 625–632). These sequences were completely conserved among 40 Japanese and 31 United States clinical isolates, whereas two United States isolates had a mutation (P570T) in the second sequence (Fig 2B). The AD-1 sequences of all isolates are available in S2 Fig.

### EV2038 affinity

**Fig 3:**
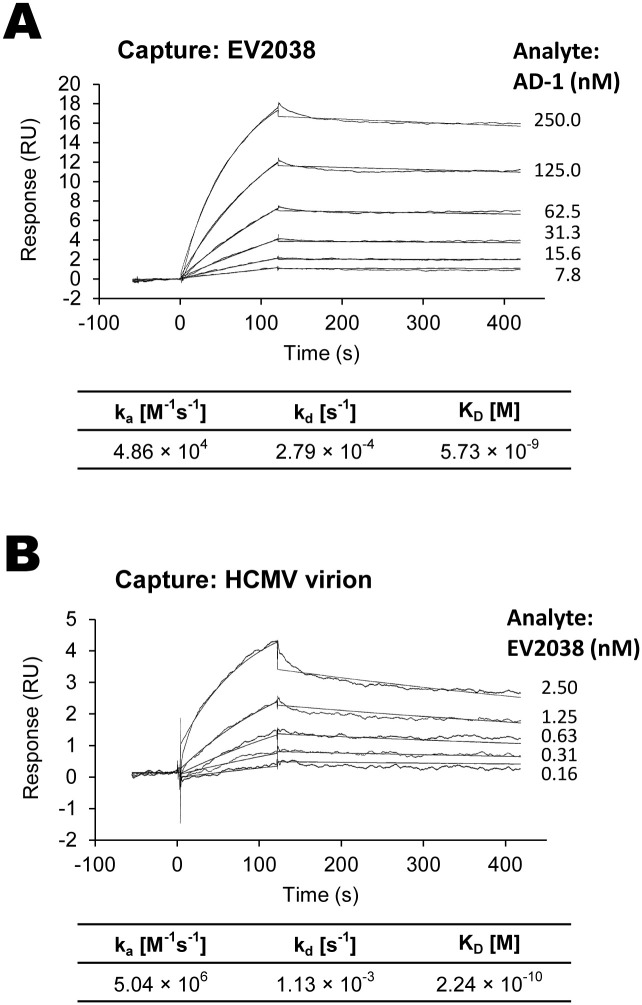
Kinetics of EV2038 binding to recombinant antigenic domain 1 and human cytomegalovirus virions. **(A)** EV2038 was captured by an anti-IgG (Fc) antibody immobilized on a sensor chip, and various concentrations of recombinant antigenic domain 1 were injected as an analyte. **(B)** Infectious virions (strain AD169, 1.6 × 10^8^ plaque-forming units/mL) were trapped by anti-human cytomegalovirus gH/gL antibody (MSL-109) captured on a sensor chip, and various concentrations of EV2038 were injected as an analyte. The kinetic parameters were the association rate constant (k_a_), dissociation rate constant (k_d_), and equilibrium dissociation constant (K_D_).

The kinetic parameters of EV2038 binding to recombinant AD-1 were calculated as an association rate constant (k_a_ value) of 4.86 × 10^4^ [mol/L]^-1^s^-1^, a dissociation rate constant (k_d_ value) of 2.79 × 10^−4^ s^-1^, and an equilibrium dissociation constant (K_D_ value) of 5.73 × 10^−9^ mol/L (Fig 3A). The kinetic parameters of EV2038 bound to HCMV virions were calculated as a k_a_ value of 5.04 × 10^6^ [mol/L]^-1^s^-1^, a k_d_ value of 1.13 × 10^−3^ s^-1^, and a K_D_ value of 2.24 × 10^−10^ mol/L (Fig 3B).

### Pharmacokinetics of EV2038

Single and multiple intravenous injections of EV2038 at different doses (0, 10, 50, and 100 mg/kg) caused no significant toxicity in either male or female cynomolgus monkeys. Death, or any clinical signs of distress to the cardiovascular, respiratory, and central nervous systems, attributable to EV2038, were not observed during the observation period (approximately 8 weeks) in any group. There were no changes related to EV2038 administration in terms of body weight, food consumption, or other parameters. Anti-EV2038 antibodies were not detected in any animal, except for one female, after a single dose of 10 mg/kg on Day 21. In the multiple-dose group that received 100 mg/kg once weekly, hematological changes (decrease in erythrocyte count, hematocrit values, and hemoglobin concentrations) were observed 7 days after the last (fourth) dose in three animals, two of which also presented with an increase in reticulocyte ratio; however, these symptoms subsided during the 4-week recovery period. Therefore, the no-observed-adverse-effect level was estimated to be 50 mg/kg/dose.

**Fig 4:**
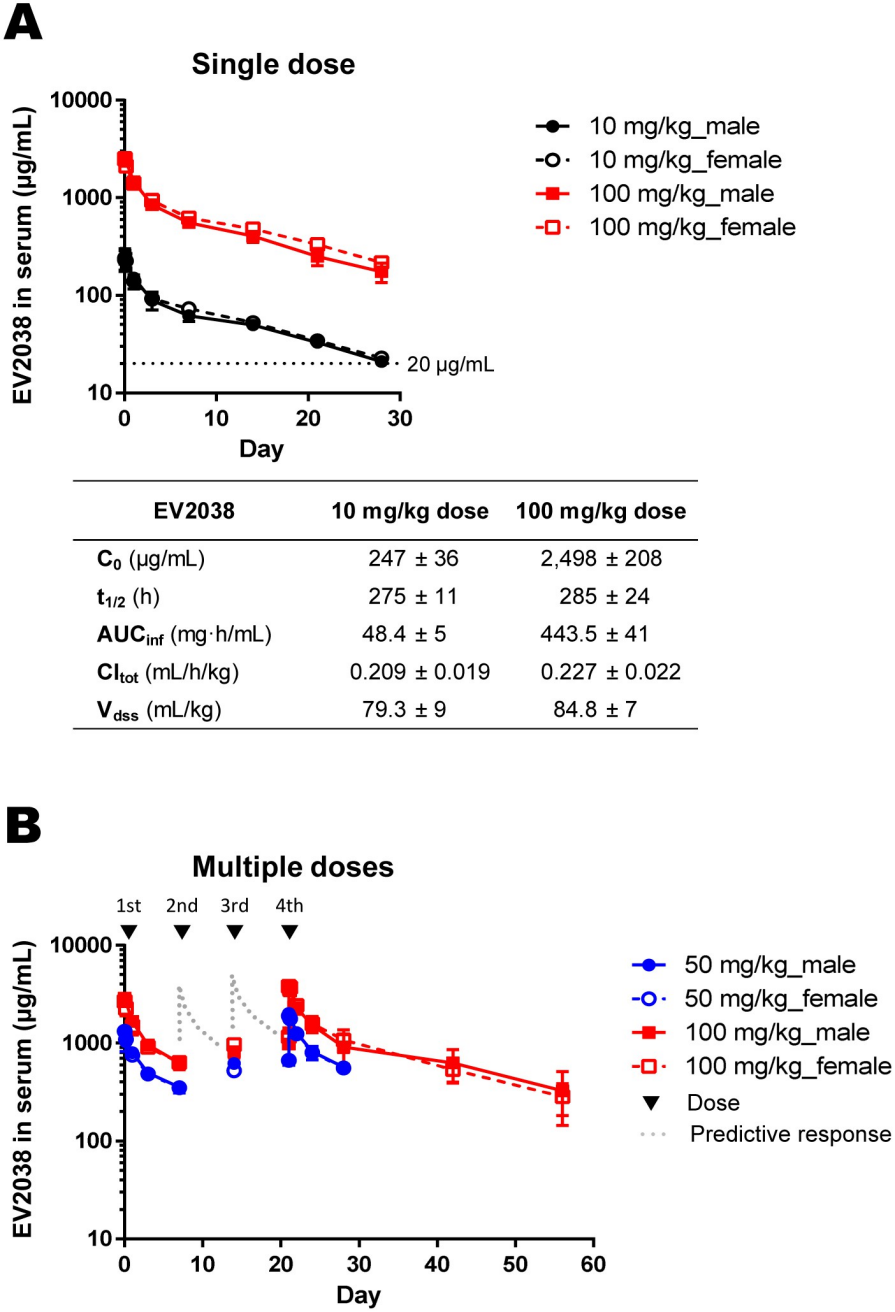
Pharmacokinetics of EV2038 in cynomolgus monkeys. **(A)** EV2038 concentrations in serum after dosing at 10 minutes, 1/4/24 hours, and 3/7/14/21/28 days in the single dose study. The pharmacokinetic parameters were the EV2038 concentration at time zero (C_0_), half-life (t_1/2_), area under the concentration–time curve (AUC) from C_0_ to infinity (AUC_inf_), total body clearance (CL_tot_), and volume of distribution at steady state (V_dss_). Data represent mean with error bars indicating the SD of two animals. **(B)** EV2038 concentrations in serum after dosing at 10 minutes, 4/24 hours, and 3/7/14/21/22/24/28/42/56 days in the multiple-dose study. Data represent mean with error bars indicating the SD of three or six animals.

There were no sex differences in pharmacokinetic parameters. EV2038 concentrations in serum increased in a dose-dependent manner after both single and multiple doses (Fig 4). The half-life of EV2038 was estimated to be between 270 and 294 hours (11.3 and 12.3 days) in the single dose study (Fig 4A). The half-life, total body clearance, and volume of distribution at steady state values were comparable between doses. EV2038 concentrations in serum were maintained at 22 and 196 μg/mL on Day 28 after a single dose of 10 and 100 mg/kg, respectively.

There was no cross-reactivity of biotinylated EV2038 between human and cynomolgus monkey tissues. No specific staining with biotinylated EV2038 at 2.5–10.0 μg/mL was observed in human or cynomolgus monkey tissues. Specific staining for EV2038 was only observed in HCMV-infected MRC-5 cells. Staining of scattered cells and diffuse staining in the luminal aspect of blood vessels were considered non-specific, because similar staining was observed with biotinylated IgG1 lambda.

## Discussion

To address the unmet need to treat HCMV disease, we isolated EV2038 (IgG1 lambda), a fully human neutralizing mAb specific to AD-1 of HCMV gB.

EV2038 is a broadly neutralizing mAb that inhibited HCMV infection of four laboratory strains and 42 Japanese clinical isolates, including ganciclovir-resistant isolates. The neutralizing titers of EV2038 against the laboratory strains were approximately 1,000 times higher than those of the anti-CMV HIG, CytoGam (Fig 1A). IC_50_ values (Fig 1B) indicate that EV2038 is comparable or over five times more potent than other clinical candidate mAbs specific to gB and gH (0.5 μg/mL in TI-23 (C23) [43], 0.31–1.27 μg/mL in TCN-202 (2N9) [44], 0.07–0.42 μg/mL in TRL345 [45], 0.28–2.79 μg/mL in LJP538 [46], 0.3–2.5 μg/mL in MSL-109 [47], 0.2–0.5 μg/mL in MCMV5322A [33], and 0.015–0.188 μg/mL in MAb 3–25 [28]), but less potent than mAbs specific to gH-pentamer (0.001–0.02 μg/mL in LJP539 [46] and 0.01–0.02 μg/mL in MCMV3068A [33]). Although mAbs targeting gH-pentamer are potent, their neutralizing activity is reduced in crucial cell types for congenital HCMV infection, including fibroblasts and trophoblast progenitor cells of the placenta, because viral entry into these cell types is dependent on gB [26, 27, 48]. Thus, the development of gB-specific mAbs, such as EV2038, is also useful to protect a large variety of cell types.

The inhibition of viral cell-to-cell spread is also an important parameter for predicting the neutralizing ability of mAbs, because *in vivo* HCMV disseminates primarily by direct cell-to-cell spread, rather than through the release of cell-free virus [49]. In a murine model of CMV infection, the ability of neutralizing mAbs to prevent cell-associated spread *in vitro* was found to correlate with their protective capacity *in vivo* [50]. However, it remains controversial which methods can accurately measure the ability of neutralizing mAbs to inhibit viral cell-to-cell spread, both *in vitro* and *in vivo* [26, 42, 51, 52]. In this study, we used a semi-solid agar overlay containing mAb to prevent the release of cell-free virus. EV2038 effectively inhibited the cell-to-cell spread of all eight clinical isolates tested in ARPE-19 cells, with IC_50_ values ranging from 1.0 to 3.1 μg/mL, and IC_90_ values ranging from 13 to 19 μg/mL (Fig 1C). Notably, the IC_90_ values of EV2038 suggest that at a dose of greater than 20 μg/mL, and administered at a relatively early stage of viral replication, EV2038 almost completely prevents subsequent rounds of HCMV infection. This potential of EV2038 may be beneficial, particularly when mAbs are used prophylactically in clinical practice.

The consistently high neutralizing activity of EV2038 against many clinical isolates suggests that the epitope recognized by EV2038 is conserved among isolates. Epitope mapping revealed that EV2038 recognizes three discontinuous sequences in AD-1 of gB (Fig 2A). Although two sequences of the epitope, at amino acids 549–560 (SCVTINQTSVKV) and 625–632 (YEYVDYLF), were newly identified in this study, the sequence of amino acids at positions 569–576 (SPGRCYSR) was similar to that previously identified at positions 570–579 [53]. The three discontinuous sequences were completely conserved among 71 clinical isolates (Fig 2B), accounting for the broadly neutralizing activity of EV2038. Among all 28 residues of the EV2038 epitope, only three residues (Y625, Y627, and F632) potentially reduce antibody binding to AD-1 by mutation [54]. This implies that HCMV is unlikely to evade EV2038 neutralization by mutating. In our preliminary study of viruses (strain AD169) grown with mAbs, the neutralization titer of EV2038 against HCMV infection barely changed (IC_50_ values ranged from 0.08 to 0.18 μg/mL) after two passages of virus, whereas MSL-109 (targeting gH/gL) significantly decreased its titer (IC_50_ values changed from 0.26 to greater than 30 μg/mL) (S3 Fig), which was probably secondary to the development of resistance through incorporation into assembling virions [55].

Recent studies revealed that the AD-1 surface is buried at the center of the prefusion gB trimer, and is completely exposed and highly accessible to non-neutralizing antibodies against post-fusion gB [19, 56–58]. Affinity analysis of EV2038 revealed effective binding to recombinant AD-1 (Fig 3A), which can be assumed to be in a stable post-fusion form. For effective neutralization of herpes virus entry, however, the ability of neutralizing antibodies to access the prefusion structure of gB before membrane fusion with host cells is important [59]. EV2038 appears to effectively bind to the AD-1 of prefusion gB, as EV2038 exhibited high affinity to infectious HCMV virions (Fig 3B), where the predominant structure of gB is in the prefusion form [58]. Therefore, we hypothesized that EV2038 can effectively bind to both prefusion and post-fusion forms of gB. This hypothesis is also supported by a mapping of the residues recognized by EV2038 in the gB structure. The epitope appears to be accessible at the center of the AD-1 surface of prefusion gB, as well as distributed on the exposed surface of AD-1 of post-fusion gB (S4 Fig). A similar antibody (SM5-1) has been reported, which binds both prefusion and post-fusion gB conformations [57]. Further structural and functional studies such as cryo-electron microscopy are needed to understand the nature of the conformational interaction between EV2038 and epitopes, and the potential mechanism of neutralization.

In the pharmacokinetics study, EV2038 was stable and safe in cynomolgus monkeys. All animals tolerated the treatment. No severe clinical signs were observed. EV2038 concentrations in monkey serum increased with dose and frequency and remained higher than 20 μg/mL until 28 days after intravenous injection of EV2038 (Fig 4). Regarding the IC_90_ values of cell-to-cell spread (Fig 1C), *in vivo* findings suggest that at least one 10 mg/kg dose of EV2038 every 28 days may be sufficient to prevent further HCMV infection. The half-life of EV2038 was estimated to be around 12 days in cynomolgus monkeys, extending up to 33 days in healthy adults, according to a phase I trial [60]. The long half-life of circulating EV2038 contributes to a reduction in the dose, and consequently, the cost of antibody therapy. Furthermore, neutralization by EV2038 in combination with antiviral agents, such as DNA polymerase inhibitors, demonstrated synergy (ganciclovir or cidofovir) and additivity (foscarnet or acyclovir) against HCMV infection *in vitro* (S5 Fig). This suggests that EV2038 may enhance the therapeutic effect of antiviral chemotherapy.

## Conclusions

We characterized a native human mAb, EV2038, and confirmed its neutralization activity against infection and cell-to-cell spread of broad strains of HCMV. Our preclinical data provide strong support for incorporating EV2038 into clinical trials. EV2038, or NPC-21 (Nobelpharma) as it is now named, is currently being tested in a phase II trial of kidney transplant recipients at high risk of HCMV infection (NCT04225923), and is considered a novel option for the treatment of HCMV disease.

## Supporting information

S1 Raw images (PDF)

S1 Fig Neutralizing activity of EV2038 with or without complements. An inhibition assay of virus infection was performed, as described previously in the Materials and methods section. Virus (strain AD169), EV2038, and complements (Cp, guinea pig serum) at final 0 or 5% (v/v) were mixed prior to infection of MRC-5 cells.
(TIF)

S2 Fig Amino acid sequence alignment of antigenic domain 1 of human cytomegalovirus among Japanese (n = 40) and United States (n = 33) clinical isolates. The predicted EV2038-binding sequences are surrounded by a frame.
(TIF)

S3 Fig Neutralizing activities of EV2038 and MSL-109 against human cytomegalovirus acclimated to antibodies. An inhibition assay of virus infection was performed, as described previously in the Materials and methods section. Virus (strain AD169) was grown for two passages **(A)** without antibody, **(B)** with EV2038 (0.2 μg/mL), or **(C)** with MSL-109 (1.8 μg/mL) before assay.
(TIF)

S4 Fig Epitope mapping of EV2038 on the surface of (A) prefusion and (B) post-fusion glycoprotein B. Residues recognized by EV2038 (S549-V560, S569-R576, and Y625-F632) are shown in magenta and antigenic domain 1 (M541-T658) in pale pink. The glycoprotein B structure is shown as a trimer with surface representation using published data (prefusion PDB ID: 7KDP; post-fusion PDB ID: 7KDD) and Pymol software (http://www.pymol.org).
(TIF)

S5 Fig Combined effects of EV2038 and antiviral agents against human cytomegalovirus infection in MRC-5 cells. An inhibition assay of virus infection was performed, as described previously in the Materials and methods section. Virus (strain AD169), 3-fold serial dilutions of EV2038 (0.00076–15 μg/mL), and 2-fold serial dilutions of anti-CMV agents targeting viral DNA polymerase (ganciclovir [GCV], 0.02–10 μg/mL; cidofovir [CDV], 0.078–40 μg/mL; foscarnet [FOS], 0.39–200 μg/mL; and acyclovir [ACV], 0.2–100 μg/mL; all from Sigma) were mixed prior to infection of MRC-5 cells. **(A)** The 50% inhibitory concentration of EV2038 at each dose was calculated using linear regression analysis. The 50% inhibitory concentration of EV2038 without anti-CMV agents was approximately 0.1 μg/mL. **(B)** The combined effects were evaluated using the Universal Response Surface Approach model of Greco et al. (Greco WR, Bravo G, Parsons JC, Pharmacol Rev. 1995 Jun;47(2):331–85. PubMed PMID: 7568331). If alpha is positive and its 95% confidence interval does not cross zero, the effect is considered synergistic. If alpha is negative and its 95% confidence interval does not cross zero, the effect is considered antagonistic. In any other case, the effect is considered additive.
(TIF)
