# Supplementary material for: A fully human neutralizing monoclonal antibody targeting a highly conserved epitope of the human cytomegalovirus glycoprotein B
Source: PLoS One. 2023 May 16;18(5):e0285672. doi: 10.1371/journal.pone.0285672 (PMC10187921; doi:10.1371/journal.pone.0285672)
Supplement: S1 Raw images — (PDF) [file pone.0285672.s001.pdf]

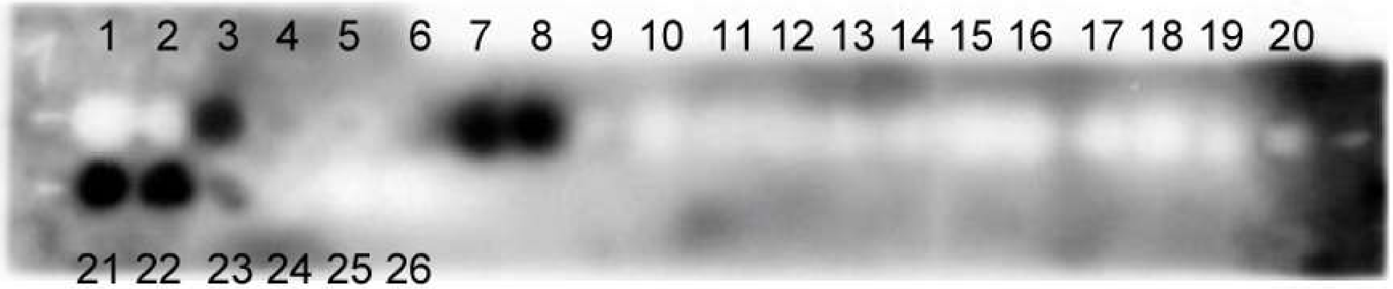

Sample: Peptides (No. 1-26) detected by EV2038 in Western blotting
Image capture: Photosensitive film exposed for 20 seconds
Figure generated: Fig 2
