# Supplementary figures and images for: A fully human neutralizing monoclonal antibody targeting a highly conserved epitope of the human cytomegalovirus glycoprotein B
Source: PLoS One. 2023 May 16;18(5):e0285672. doi: 10.1371/journal.pone.0285672 (PMC10187921; doi:10.1371/journal.pone.0285672)

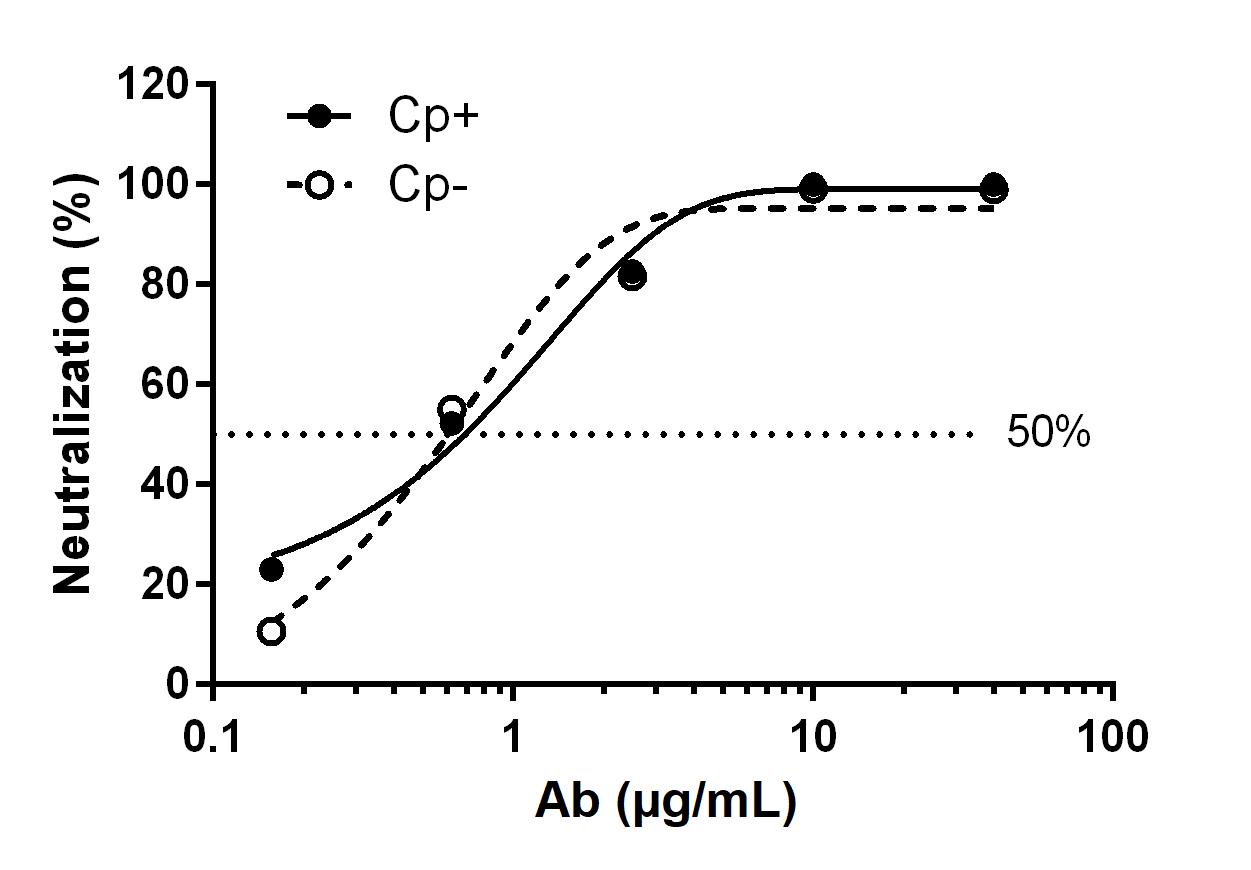

Supplement: S1 Fig — An inhibition assay of virus infection was performed, as described previously in the Materials and methods section. Virus (strain AD169), EV2038, and complements (Cp, guinea pig serum) at final 0 or 5% (v/v) were mixed prior to infection of MRC-5 cells. (TIF) [file pone.0285672.s002.tif]

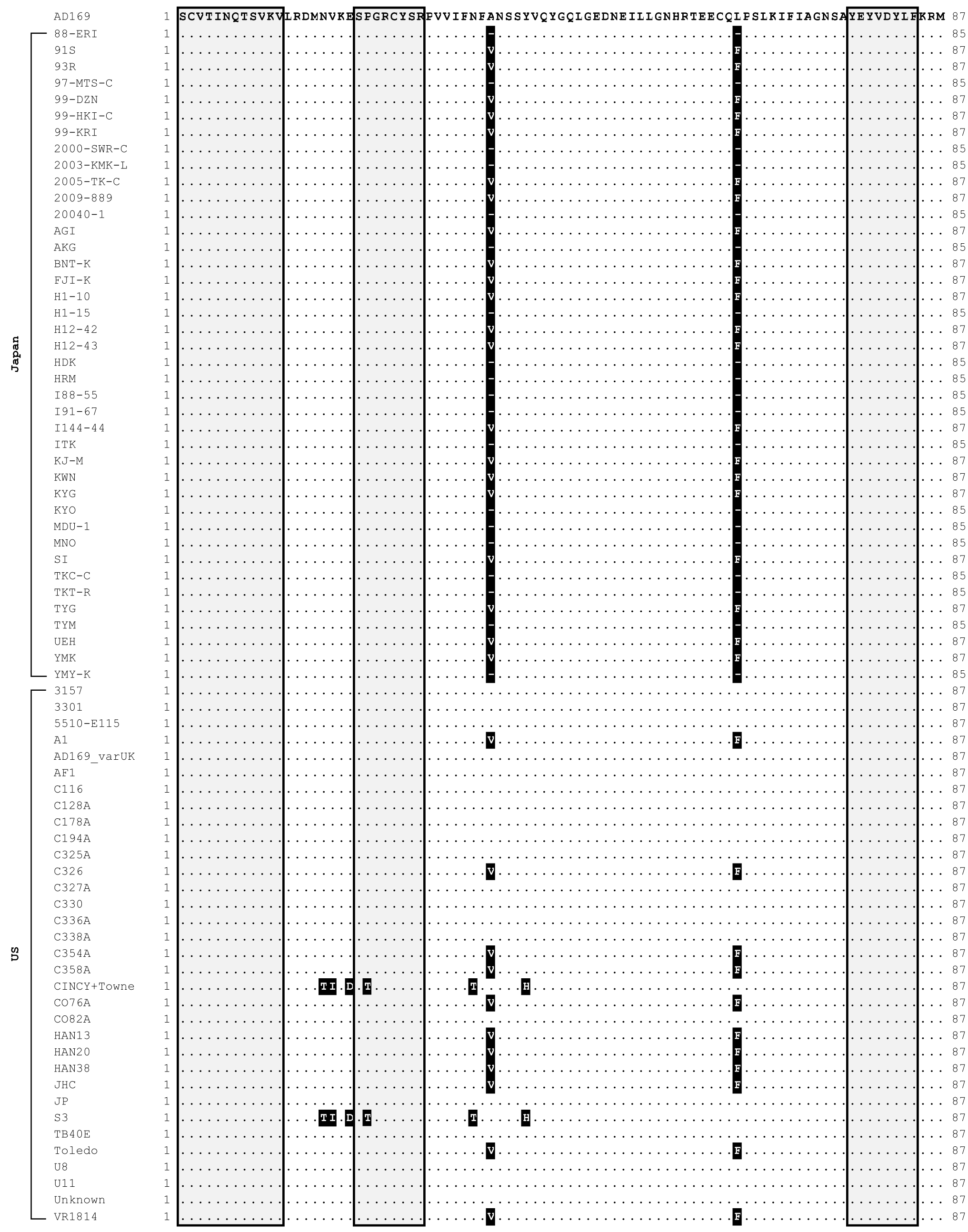

Supplement: S2 Fig — The predicted EV2038-binding sequences are surrounded by a frame. (TIF) [file pone.0285672.s003.tif]

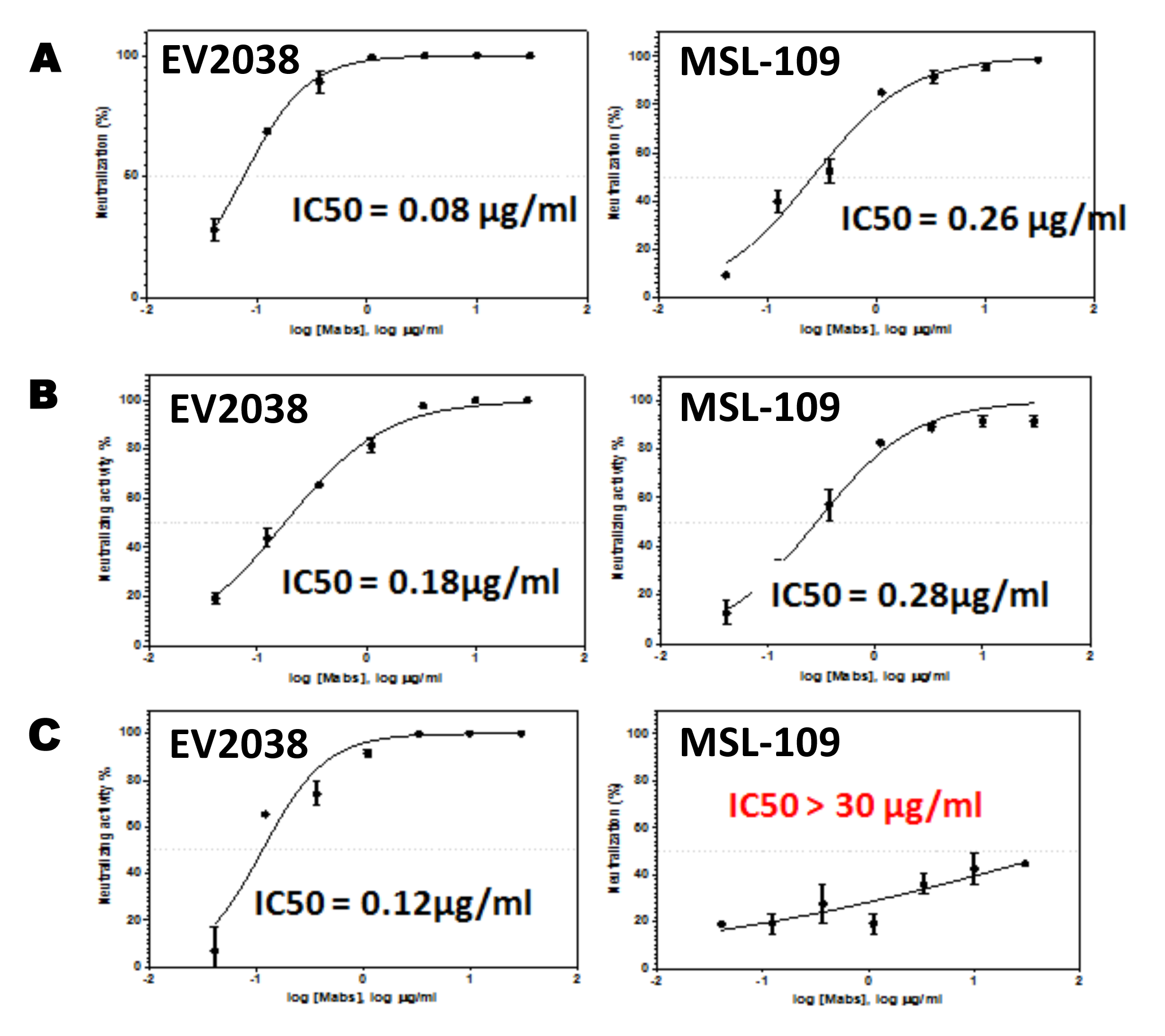

Supplement: S3 Fig — An inhibition assay of virus infection was performed, as described previously in the Materials and methods section. Virus (strain AD169) was grown for two passages (A) without antibody, (B) with EV2038 (0.2 μg/mL), or (C) with MSL-109 (1.8 μg/mL) before assay. (TIF) [file pone.0285672.s004.tif]

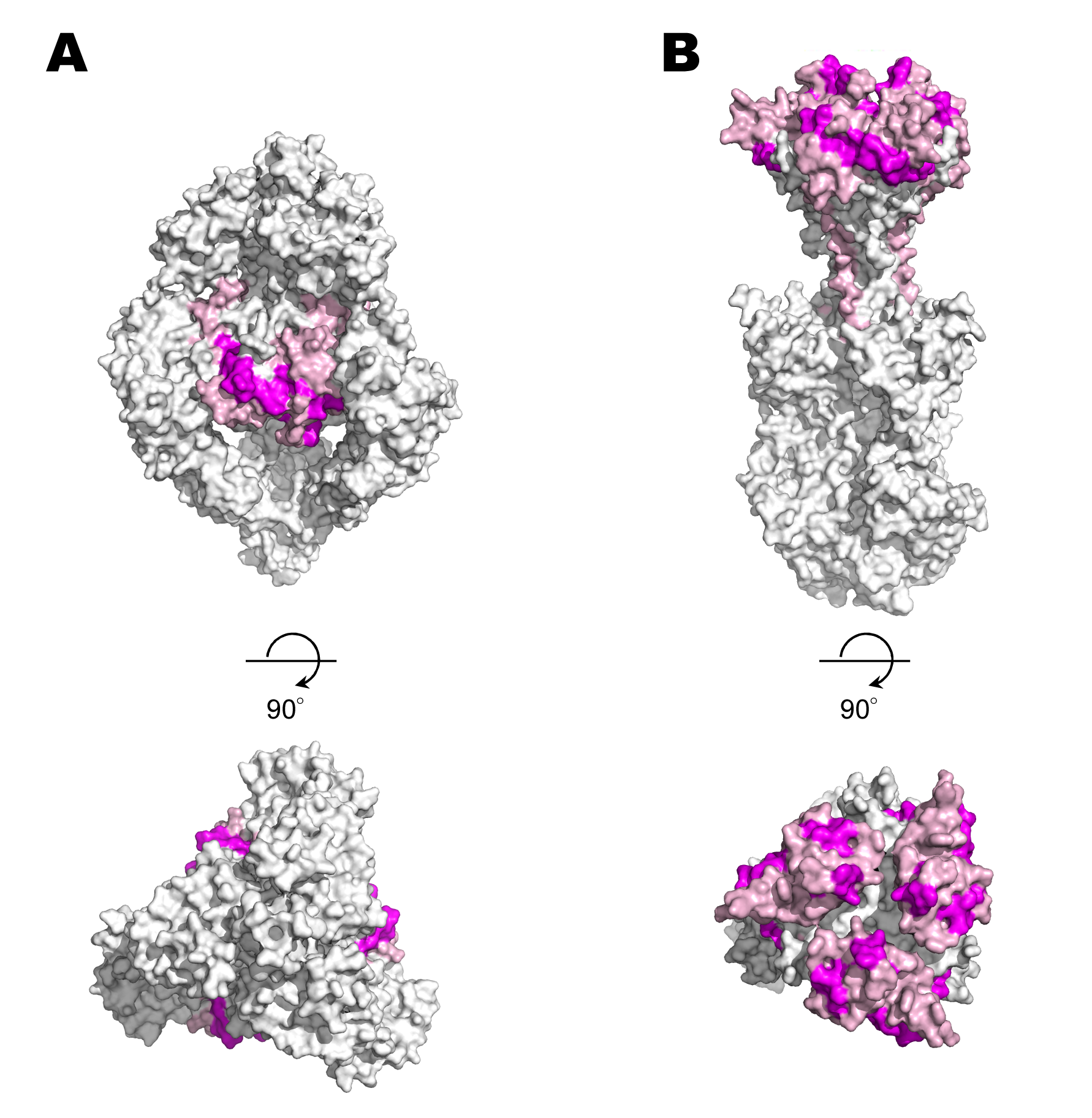

Supplement: S4 Fig — Residues recognized by EV2038 (S549-V560, S569-R576, and Y625-F632) are shown in magenta and antigenic domain 1 (M541-T658) in pale pink. The glycoprotein B structure is shown as a trimer with surface representation using published data (prefusion PDB ID: 7KDP; post-fusion PDB ID: 7KDD) and Pymol software (http://www.pymol.org). (TIF) [file pone.0285672.s005.tif]

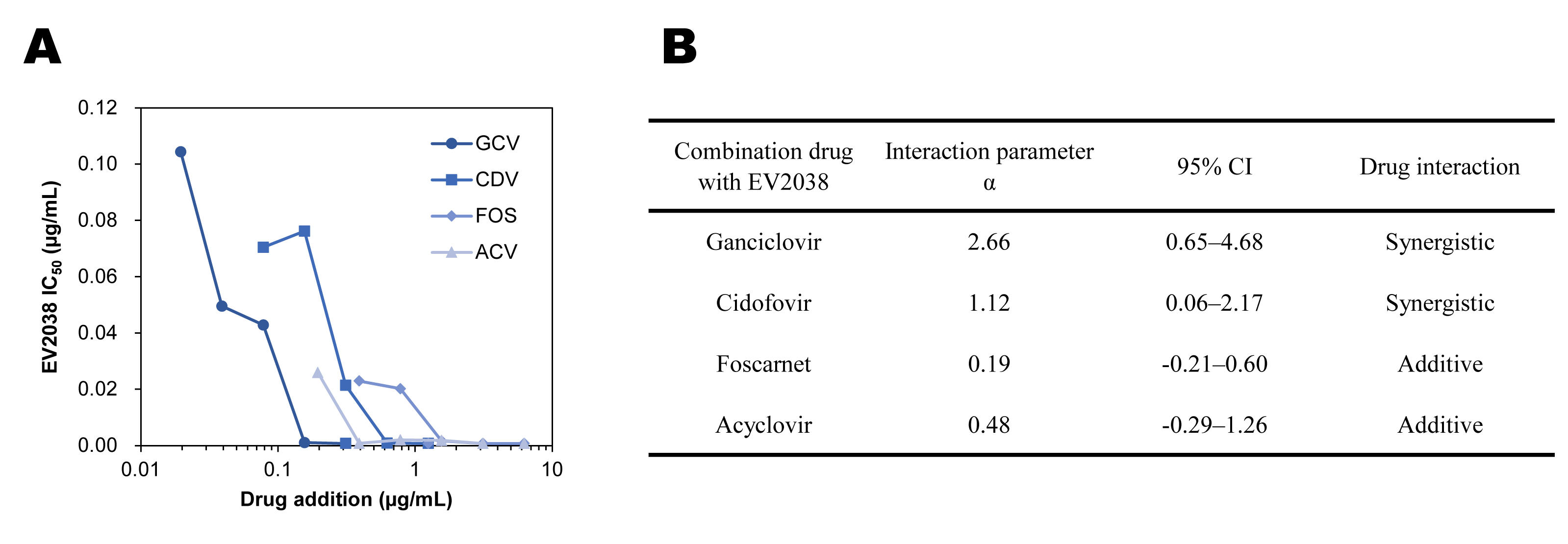

Supplement: S5 Fig — An inhibition assay of virus infection was performed, as described previously in the Materials and methods section. Virus (strain AD169), 3-fold serial dilutions of EV2038 (0.00076–15 μg/mL), and 2-fold serial dilutions of anti-CMV agents targeting viral DNA polymerase (ganciclovir [GCV], 0.02–10 μg/mL; cidofovir [CDV], 0.078–40 μg/mL; foscarnet [FOS], 0.39–200 μg/mL; and acyclovir [ACV], 0.2–100 μg/mL; all from Sigma) were mixed prior to infection of MRC-5 cells. (A) The 50% inhibitory concentration of EV2038 at each dose was calculated using linear regression analysis. The 50% inhibitory concentration of EV2038 without anti-CMV agents was approximately 0.1 μg/mL. (B) The combined effects were evaluated using the Universal Response Surface Approach model of Greco et al. (Greco WR, Bravo G, Parsons JC, Pharmacol Rev. 1995 Jun;47(2):331–85. PubMed PMID: 7568331). If alpha is positive and its 95% confidence interval does not cross zero, the effect is considered synergistic. If alpha is negative and its 95% confidence interval does not cross zero, the effect is considered antagonistic. In any other case, the effect is considered additive. (TIF) [file pone.0285672.s006.tif]
